# Report on the Relation of the Druggist to the Physician

**Published:** 1871-12

**Authors:** H. N. Rosser


### A REPORT ON "THE RELATION OF THE DRUGGIST TO THE PHYSICIAN."

*Read at the Semi-Annual Meeting of the Dallas County Medical Association, held at Selma, Ala., November 14th, 1871, by H. N. Rosser. M. D.*

The time that has been allotted for me to prepare an essay to be read betore this Society has, this evening, expired; and as, in my opinion, it is the duty of every member to contribute his mite, whether it be of interest or not, I submit this report.

The subject selected for me should, of all others, claim at this date of advancement in science, the closest attention by every medical man.

Practical pharmacy, whether it is from a want of a knowledge of the workings of pharmaceutical establishments or an apathy on the part of the practitioner, I am not prepared to say or discuss, yet it is so plain, "that he who runs may read," that our apothecary establishments are not conducted in a manner in keeping with the spirit of the times or the advance of science. Chemistry, either organic or inorganic, is the basis of all pharmacuetical preparations. Yet it is of the rarest occurrence to find an apothecary with sufficient chemical knowledge to discuss the most simple formula which may be submitted to him. And at the first glance it would appear that there is no necessity for a general acquaintance with those laws upon which the superstructure of his business is founded, if we judge from the total ignorance of those who take upon themselves the compounding of remedies with which we are wont to combat disease.

In the relationship existing between the physician and apothecary there should be an implicit confidence on the part of the physician; and with that knowledge of the business which should be possessed by every practical druggist, there should be an honesty of purpose always to detect fraud; furnishing the doctor with the most select drugs and pharmaceuticals which can be procured. Some years since, I was very much amused at a practitioner of medicine who had never seen proper to take that elegant aperient, magnesia cit. Our "medica" was unwell, and called for a bottle of said aperient; the proprietor of the establishment waited upon him, and administered a dose which he pronounced fresh and genuine. Our good doctor unsuspectingly swallowed the draught; but next morning complained of a most disagreeable action of the solution, and expressed his determination of never using the sol. of cit. magnesia in his practice. I subjoin the formula by which the medicine was prepared, and you will not be surprised at the action of the remedy:

R.—Sodæ Carb.;

Acid Tart.;

Syrup Simp.;

Tinct. Ol. Lemon;

Water.

Make a solution, and add potass. bicarb., a sufficient quantity to each bottle, cork tightly. Thus you see how an elegant preparation has been condensed by the cupidity of the druggist. I simply mention this instance to show the importance of the physician in his selection of a druggist to have an implicit confidence in his integrity, as well as his acquirements.

There should be a courtesy and over-looking disposition cultivated by the physician toward the apothecary. After selecting that apothecary in whom you have implicit confidence, you should recollect that there are many little errors committed, which of themselves are of no danger, nor do they show a want of a knowledge of the business; yet, if made known, would materially injure the reputation of the pharmaceutist. These should be shielded as far as possible, and if the one who committed the error be an employee, he should be made known of his error privately, so that he may be more careful in the future, for we, as practitioners of medicine, are constantly making errors of the gravest nature which, if it were not for the vigilance of the apothecary, would produce very serious results. Yet I have observed that those who make the most serious blunders are the first to condemn the apothecary for any misstep he may make. I do not wish to be understood that I advocate shielding ignorance or carelessness, far be it from me. I am one of the first to cry it down, still there is a forbearance which should be had in moderation, and circumstances should be taken into consideration which, if known, would materially modify our action.

If our friends of the spatula and mortar would have our support, they should employ experienced help, which, I am sorry to say, is not always the case. During the past five years there have been errors of the most culpable nature in our little central city, originating entirely from the ignorance of the apothecary; and this is not only the case in Selma, but everywhere that I have known of the workings of drug stores where incompetent young men were employed. Thus it is that we so often find our remedies of such variable strengths and therapeutic action, and on account of which we are misguided, often giving heroic doses at one time, and at others, finding that homeopathic portions have the desired effect. Fortunately for the apothecary, physicians have a reason for everything; and a peculiar idiosyncrasy of the patient is blamed for the inertia or too powerful action of remedies, when, if we were to look further, we would find a fault in preparation and not in "Dame Nature."

Substitutions by some druggists is practiced in no small degree; for instance, using Ext. of Taraxacum instead of Ext. of Gentian, as an excipient for pills, two remedies widely different in their remedial action; such substitutions are often made from ignorance of the therapeutic action of remedies; but more often from an indisposition to procure such articles as are required, and occasionally from a desire for pecuniary gain. An example of this kind has already been cited. All such customs, of course, are condemned by the intelligent practitioner, and under no circumstances should they be permitted without first consulting the physician.

In our dealings with apothecaries, some of us are very much disposed to be in too great a hurry with our chirography. Doctors, as well as lawyers, are proverbial for their illegible writing. Yet the physician, of all others, should be the most careful in writing his prescriptions or orders, if he expects them compounded or executed carefully.

There is a custom amongst most apothecaries which can not be too highly censured by the practitioner of medicine. I have reference to using the physician's prescription in the treatment of any case which he may have presented to him. Still I would not wish to condemn the druggist in selling his medicines which may be on sale, such as patent medicines. Yet, to diagnose a case and prescribe for the patient, by using the prescriptions which may be intrusted to his skill for preparation by the physician, is a betrayal of trust and a species of injustice which should not be tolerated.

Volumes might be written concerning the interior workings of drug stores which directly affects the physician in his success, that can only be remedied by law, which is done in most enlightened countries; and if we wish to know what we give our patients, we should so endeavor to get the Legislature of our State to govern, by enactment, all pharmaceutical establishments. There is such an invitation to fraud that we can hardly expect to get those remedies which are of undoubted purity, until there shall be some legal restraint upon the compounding of drugs. The medical societies of other States are moving in this direction, and I hope it will not be long before the physicians of Alabama may see the benefit accruing from a properly licensed pharmaceutical system.

